# Revised diagnosis of the flathead genus *Elates* (Teleostei, Platycephalidae) and description of a new species collected from a fish market near Sarangani Bay, Philippines

**DOI:** 10.3897/zookeys.1281.181028

**Published:** 2026-06-01

**Authors:** Ned S. Rose, Maybelle A. Fortaleza, Joey P. Cabasan, Kevin L. Labrador, Joemarie J. Lanutan, Cleto L. Nañola Jr., Matthew G. Girard, Katherine E. Bemis

**Affiliations:** 1 Department of Vertebrate Zoology, National Museum of Natural History, Smithsonian Institution, Washington, DC 20560, USA Department of Biology, University of Antwerp Antwerp Belgium; 2 National Systematics Laboratory, Office of Science and Technology, NOAA Fisheries, Washington, DC 20560, USA Coral Reef Resiliency and Ecology Studies Laboratory, University of the Philippines Davao Philippines; 3 Department of Fish and Wildlife Conservation, College of Natural Resources and Environment, Virginia Polytechnic Institute and State University, VA 24060, Blacksburg, USA Department of Life Sciences, Texas A&M University - Corpus Christi Corpus Christi United States of America; 4 Coral Reef Resiliency and Ecology Studies Laboratory, University of the Philippines, Mindanao, Davao City 8000, Philippines Department of Vertebrate Zoology, National Museum of Natural History, Smithsonian Institution Washington, DC United States of America; 5 Department of Biology, University of Antwerp, 2018 Antwerp, Belgium Department of Fish and Wildlife Conservation, College of Natural Resources and Environment, Virginia Polytechnic Institute and State University Blacksburg United States of America; 6 Department of Life Sciences, Texas A&M University - Corpus Christi, Corpus Christi, TX 78412, USA National Systematics Laboratory, Office of Science and Technology, NOAA Fisheries Washington, DC United States of America

**Keywords:** Biodiversity, COI, General Santos, ichthyology, Mindanao, mitochondrial genome, taxonomy

## Abstract

We revise the diagnosis of the flathead genus *Elates* Jordan & Seale, 1907 and describe a new species based on specimens purchased from a fish market in the southern Philippines. The new species differs from the only previously valid species in the genus, *Elates ransonnettii* (Steindachner, 1876), in having a large eye, a broad lateral vomerine tooth patch, a medial vomerine tooth patch, two pores on each lateral-line scale, a greater number of scale rows above the lateral line, an absence of a filamentous ray extending from the upper lobe of the caudal fin, a transparent dorsal-fin membrane with no dark pigmentation, and a wholly dark peritoneum. Analysis of cytochrome *c* oxidase subunit I (COI) data supports both genetic differentiation between *E. ransonnettii* and the new species as well as monophyly of the genus *Elates*. The new species is known only from specimens purchased in the General Santos Public Market in General Santos City caught in Sarangani Bay, in contrast to *E. ransonnettii*, which is broadly distributed in the western Pacific Ocean to the Malacca Strait and Andaman Sea.

## Abstract (es)

Aming muling sinuri ang mga isdang kabilang sa genus *Elates*, na kilalang isdang-buwaya o isdambuwaya sa Tagalog, at inilarawan ang isang bagong species batay sa labing-walong indibidiwal na nabili sa pamilihang-isda sa katimugang Pilipinas. Ang bagong species na ito ay kahawig ng *E. ransonnettii*, na karaniwang tinatawag na “sunogan” sa Bisaya. Ang bagong species ay naiiba sa pagkakaroon ng malapad na lateral vomerine tooth patch, isang medial vomerine tooth patch, malaking mata, dalawang butas sa bawat kaliskis sa gitnang bahagi ng katawan (lateral-line scales), mas maraming hanay ng kaliskis sa ibabaw ng linya sa gitnang bahagi ng katawan (number of scale rows above lateral line), malinaw na lamad ng dorsal na palikpik, maitim na peritoneum, at kawalan ng filamentous ray sa bahaging itaas ng buntot. Ang pagkakaiba ng bagong species sa *E. ransonnettii* ay nabigyang-diin sa pamamagitan ng pagsuri ng cytochrome *c* oxidase I (COI) sequences, at kinumpirma din nito ang monophyly ng genus *Elates*. Ang bagong species ay kasalukuyang kilala lamang sa Sarangani Bay, na taliwas sa malawak na distribusyon ng *E. ransonnettii*.

## Introduction

The Platycephalidae, or flatheads, is a family of benthic fishes primarily distributed throughout the tropical and temperate Indo-Pacific waters, with one species native to the eastern Atlantic Ocean (Imamura 1996). Some species, such as *Platycephalus fuscus* Cuvier, 1829, support important commercial trawling and recreational fisheries (Gray and Barnes 2015). The most recent and comprehensive analyses of morphological (Imamura 1996) and molecular (Puckridge et al. 2019) characters recover the family Platycephalidae as comprising two subfamilies: the Onigociinae and the Platycephalinae. The more diverse Onigociinae includes 15 genera and 79 species (Imamura 1996; Fricke et al. 2025), compared to the Platycephalinae that includes only two genera: *Platycephalus* Bloch, 1795, with 18 species, and the monotypic genus *Elates* Jordan & Seale, 1907 (Imamura 1996; Fricke et al. 2025). However, there are many undescribed species of platycephalids, as shown by Puckridge et al. (2012), who examined 49 species of platycephalids but recognized 65 operational taxonomic units. Although both Keenan (1991), using allozymes, and Imamura (1996), using morphological characters, recognized *Elates* and *Platycephalus* to be separate sister lineages, the molecular phylogeny by Puckridge et al. (2019) recovered the genus *Elates* nested within *Platycephalus*. Thus, additional taxonomic study of Platycephalidae at both the species and higher levels are needed.

The genus *Elates* was described as having an elongate upper caudal-fin lobe, small scales, and a single preopercular spine (Jordan and Seale 1907). Imamura (1996) diagnosed the genus based on its narrow head and body, two suborbital spines present below the middle and posterior section of the eye, two vomerine tooth patches (= prevomer in Imamura 1996), a single long spine on the preopercle, one posterior opening on each lateral-line scale, no interopercular flap, and undeveloped sensory “tubes” on the cheek (Imamura 1996: 200). Imamura (1996) also recognized two characters unique to *Elates* not included in the diagnosis for the genus: the absence of a coronomeckelian bone and the fusion of the third preural centrum with the hemal spine. One species, *Elates ransonnettii* (Steindachner, 1876), is currently recognized, with two available names considered junior synonyms. *Hyalorhynchus pellucidus* Ogilby, 1910 was placed in synonymy with *E. thompsoni* Jordan & Seale, 1907 by McCulloch (1930). *Elates thompsoni*, the type species of *Elates*, was placed in synonymy with *Platycephalus ransonnettii* (Steindachner, 1876) by Paxton et al. (1989). Thus, the name *Elates* was retained, making *E. ransonnettii* the valid senior synonym. *Elates ransonnettii* is primarily distributed in the western Pacific Ocean (Knapp 1999) with some occurrences in the Malacca Strait and Andaman Sea (USNM 345443, USNM 445018, USNM 445097, and USNM 445082), but it has also been introduced in the Mediterranean and Adriatic Seas (Mastrototaro 2007; Dulčić 2010). Specimens of *E. ransonnettii* have been collected from depths of 5–53 m and are generally found in sandy or muddy habitats (Knapp 1999). The species is frequently taken by trawls, occurring in 66.7% of night trawls in the Gulf of Carpentaria (Blaber et al. 1994). The largest reported individuals are about 19 cm in total length (Knapp 1999).

**Figure 1.**
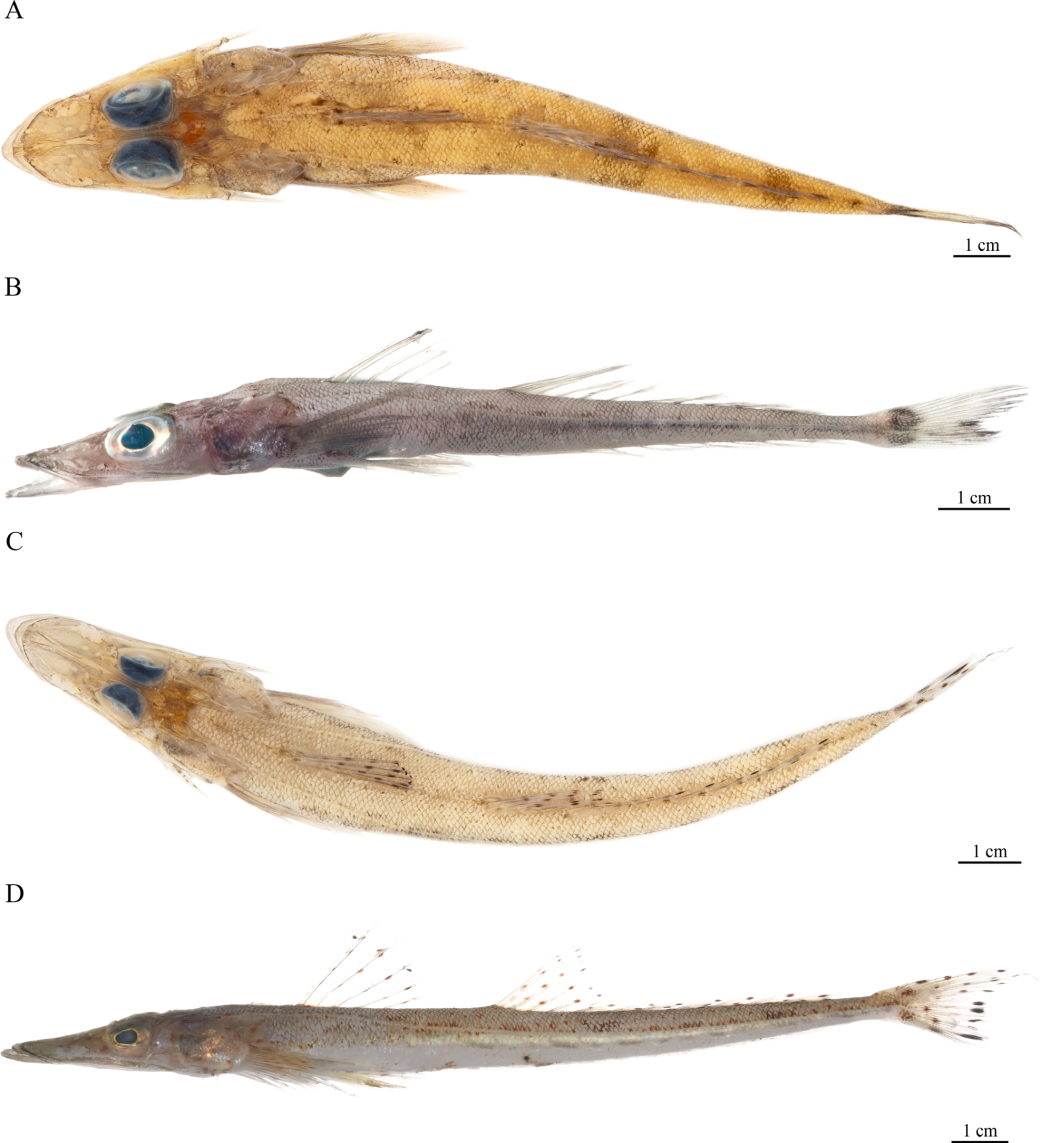
*Elates* species. **A**. *E. saranganiensis* sp. nov., dorsal view, preserved holotype (UPMIN FDP_SGES_2208_090, 151.4 mm SL); **B**. *E. saranganiensis* sp. nov., lateral view, fresh paratype (USNM 408876, 124.2 mm SL); **C**. *E. ransonnettii*, dorsal view, fresh specimen (USNM 477242, 156.0 mm SL); **D**. *E. ransonnettii*, lateral view, preserved specimen (USNM 424737, 138.0 mm SL). Note the dark pigment spots present in dorsal fins of *E. ransonnettii* that are absent in *E. saranganiensis* sp. nov.

A collaboration between the United States Food and Drug Administration, Bureau of Fisheries and Aquatic Resources – National Fisheries Research and Development Institute, Department of Agriculture, Philippines, and the National Museum of Natural History, Smithsonian Institution supported fish market collections throughout the Philippines from 2011 to 2019. The purpose of these collections was to build a genetic barcode reference library to support seafood safety and enhance biodiversity knowledge (Bemis et al. 2023). To date, six new species have been described using material from these collections (e.g. Smith-Vaniz and Johnson 2016; Matsunuma et al. 2017; Girard et al. 2024). In 2012, a specimen of an unknown platycephalid morphologically similar to but genetically distinct from *E. ransonnettii* was purchased from the General Santos Public Market in General Santos City. Vendors reported that it was captured from the vicinity of Glan, Sarangani (USNM 408876; Fig. 1). Seventeen more specimens of this platycephalid were purchased by the Coral Reef Resiliency and Ecology Studies Laboratory from the same location in 2022 (UPMIN FDP_SGES_2112_020A, UPMIN FDP_SGES_2208_088 to UPMIN FDP_SGES_2208_090) and in 2024 (UPMIN FDP_SGES_2407_80A to UPMIN FDP_SGES_2407_80M), captured from the vicinity of Buayan, General Santos City. Further examination of these specimens revealed them to be distinct from *E. ransonnettii*, but fitting the diagnosis of the monotypic *Elates* better than any other flathead genus (Imamura 1996). Herein, we describe this new species, place it in phylogenetic context, discuss its natural history, and re-diagnose the genus *Elates* in light of the newly described species.

## Materials and methods

### Specimen examination

Measurements follow Hubbs and Lagler (1958), with standard length (SL) following the fisheries-based methodology. Caudal-peduncle length was measured as the length from the posterior-most anal-fin base to the posterior end of the caudal peduncle. Nomenclature of head spines follows Knapp et al. (2000). Descriptions of lateral-line scales follow Imamura (1996). We follow Motomura (2004) for counts of longitudinal-scale rows, equating this count to the “scale rows above lateral line slanting downward and backward” used by Imamura and Knapp (2009: Table 1). To examine the coloration of the peritoneum, we opened the abdominal cavity of one specimen (USNM 408876) of the new species and 34 specimens of *E. ransonnettii*. While examining these specimens, we noted sex and maturity. The end of the upper lobe of the caudal fin was inspected for the presence of a filamentous ray in each specimen. We present data for the description based on the holotype with data for paratypes provided in parentheses when different from the holotype. See Table 1 for comparative material examined. Standard institutional codes follow those listed by Sabaj (2020). The holotype is accessioned at the University of the Philippines Mindanao, Davao City, Philippines (**UPMIN**) and paratypes are accessioned at UPMIN and the National Museum of Natural History, Smithsonian Institution, Washington, DC, USA (**USNM**).

**Table 1.** Selected comparative material examined. “*” indicate specimens used in Table 3.

Species	Catalog no.	Count	SL (mm)	Locality	Date
*Elates ransonnettii*	USNM 424737*	1	138	Philippines	09 Jun. 2013
*Elates ransonnettii*	USNM 437616*	1	137	Philippines	14 Jul. 2015
*Elates ransonnettii*	USNM 477242*	1	156	Vietnam	29 Jan. 2024
*Elates ransonnettii*	USNM 447065	57	76–156	Indonesia	5 Dec. 1975
*Elates ransonnettii*	USNM 445119	3	115–135	Philippines	5 Jun. 1978
*Elates ransonnettii*	USNM 445105	2	116–142	Philippines	Oct. 1979
*Elates ransonnettii*	USNM 445133*	27	137–152	Philippines	Feb. 1980
*Elates ransonnettii*	USNM 264801	2	133–149	Indonesia	29 May 1983
*Elates ransonnettii*	USNM 212202	1	90	Papua New Guinea	2 Dec. 1970
*Elates ransonnettii*	USNM 99705	1	119	Philippines	NA
*Elates ransonnettii*	USNM 340586	1	171	China	Oct. 1965
*Elates ransonnettii*	USNM 345443	1	115	Myanmar	17 Jul. 1996
*Elates ransonnettii*	USNM 340480	1	138	Philippines	30 Sep. 1995
*Elates ransonnettii*	USNM 99018	1	69	Philippines	10 May 1909
*Elates ransonnettii*	USNM 447193	6	107–139	Philippines	3 Mar. 1961
*Elates ransonnettii*	USNM 445018	5	133–154	Thailand	10 Feb. 1966
*Elates ransonnettii*	USNM 99653*	9	83–128	Philippines	8 Feb. 1909
*Elates ransonnettii*	USNM 99704	8	87–114	Philippines	10 May 1909
*Elates ransonnettii*	USNM 445014	1	129	Philippines	17 May 1969
*Elates ransonnettii*	USNM 444994	1	131	Philippines	30 Apr. 1980
*Elates ransonnettii*	USNM 445013	1	NA	Philippines	10 May 1909
*Elates ransonnettii*	USNM 445001	1	61	Philippines	9 Jun. 1978
*Elates ransonnettii*	USNM 445097	10	52–113	Malaysia	6 May 1969
*Elates ransonnettii*	USNM 445110	2	139–144	Papua New Guinea	7 Jun. 1979
*Elates ransonnettii*	USNM 445082	2	63–84	Malaysia	6 May 1969
*Elates thompsoni*	USNM 53068	1	136	Philippines	1907
*Platycephalus indicus*	USNM 437974	1	146	Philippines	22 Jul. 2015
*Platycephalus indicus*	USNM 437972	1	147	Philippines	22 Jul. 2015

### Imaging

Specimens were photographed in the field to capture life-like colors by J.T. Williams using Fujifilm and Nikon camera bodies with 105 mm macro lens under flash or LED daylight lighting (Bemis et al. 2023). To photograph preserved specimens, we used a Nikon D850 with 105 mm macro lens and a Sony a6500 with a 90 mm macro lens. To photograph the anatomy of lateral-line scales, we used a dissecting microscope and SwiftCam SC2003R-FL microscope camera (Swift Optical Instruments). We removed several scales to stain with alizarin red and photograph under high-energy royal-blue lights (440–460 nm) following Smith et al. (2018), with filter and LED-light modifications of Girard et al. (2020, 2022). To examine internal osteology, we generated µCT scans using a GE Phoenix v|tome|x M 240/180kV Dual Tube µCT scanner at the National Museum of Natural History, Smithsonian Institution. These scans are publicly available on MorphoSource (Table 2). We used 3D Slicer v. 5.8.1 (Rolfe et al. 2021) and the SlicerMorph module (Fedorov et al. 2012) to isolate and manipulate the lighting and opacity of µCT scan data.

**Table 2.** Genetic and microcomputed tomography information for specimens used in this study. “*” indicates whole annotated mitochondrial genome.

Species	Catalog no.	GenBank no.	BOLD no.	MorphoSource ID
*Elates saranganiensis* sp. nov.	UPMIN FDP_SGES_2208_088	PZ024375*	NA	NA
*Elates saranganiensis* sp. nov.	UPMIN FDP_SGES_2208_089	PZ024374*	NA	NA
*Elates saranganiensis* sp. nov.	UPMIN FDP_SGES_2208_090	PZ024373*	NA	NA
*Elates saranganiensis* sp. nov.	USNM 408876	PZ205242	PHILA550-13	000817166
*Elates saranganiensis* sp. nov.	UPMIN FDP_SGES_2407_80A	PX978870	GBAAZ69902-26	NA
*Elates saranganiensis* sp. nov.	UPMIN FDP_SGES_2407_80B	PX978869	GBAAZ70517-26	NA
*Elates saranganiensis* sp. nov.	UPMIN FDP_SGES_2407_80C	PX978868	GBAAZ69965-26	NA
*Elates saranganiensis* sp. nov.	UPMIN FDP_SGES_2407_80D	PX978867	GBAAZ69218-26	NA
*Elates saranganiensis* sp. nov.	UPMIN FDP_SGES_2407_80E	PX978866	GBAAZ71106-26	NA
*Elates saranganiensis* sp. nov.	UPMIN FDP_SGES_2407_080F	PX978865	GBAAZ69198-26	NA
*Elates saranganiensis* sp. nov.	UPMIN FDP_SGES_2407_80G	PX978864	GBAAZ69121-26	NA
*Elates saranganiensis* sp. nov.	UPMIN FDP_SGES_2407_80H	PX978863	GBAAZ69440-26	NA
*Elates saranganiensis* sp. nov.	UPMIN FDP_SGES_2407_80I	PX978862	GBAAZ69196-26	NA
*Elates saranganiensis* sp. nov.	UPMIN FDP_SGES_2407_80J	PX978861	GBAAZ69394-26	NA
*Elates saranganiensis* sp. nov.	UPMIN FDP_SGES_2407_80K	PX978860	GBAAZ70755-26	NA
*Elates saranganiensis* sp. nov.	UPMIN FDP_SGES_2407_80L	PX978859	GBAAZ69313-26	NA
*Elates saranganiensis* sp. nov.	UPMIN FDP_SGES_2407_80M	PX978858	GBAAZ69843-26	NA
*Elates saranganiensis* sp. nov.	UPMIN FDP_SGES_2112_020A	OR524370.1	MINDA335-23	NA
*Elates ransonnettii*	USNM 437616	OQ386000.1	PHILA1643-16	000817169
*Elates ransonnettii*	UMT EEZ450	OR918574.1	DBEEZ078-23	NA
*Elates ransonnettii*	CSIRO H 6710-02	GU673226.1	FOAH154-08	NA
*Platycephalus bassensis*	CSIRO BW-A9441	JN312819.1	FOAL1144-10	NA
*Platycephalus grandispinis*	CSIRO BW-A8440	HM902692.1	FOAK647-10	NA
*Platycephalus laevigatus*	CSIRO BW-A521	DQ107980.1	FOA521-04	NA
*Platycephalus caeruleopunctatus*	CSIRO BW-A8413	HM902668.1	FOAK620-10	NA
*Platycephalus speculator*	CSIRO BW-A541	DQ107952.1	FOA541-04	NA
*Platycephalus orbitalis*	CSIRO H 6381-03	JX488284.1	FOAF384-07	NA
*Platycephalus marmoratus*	CSIRO BW-A536	DQ107963.1	FOA536-04	NA
*Platycephalus indicus*	USNM 437972	NA	PHILA1999-16	NA
*Platycephalus endrachtensis*	CSIRO BW-A491	DQ107998.1	FOA491-04	NA
*Platycephalus conatus*	NMV A25185-002	JX488183.1	FMVIC1014-08	NA
*Suggrundus macracanthus*	USNM 424659	OQ386569.1	PHILA1048-13	NA
*Sunagocia otaitensis*	USNM 443453	OQ385919.1	PHILA2804-18	NA
*Hoplichthys haswelli*	CSIRO BW-A3242	NA	FOAF370-07	NA
*Scorpaenopsis cotticeps*	USNM 432588	OQ385562.1	PHILA1587-15	NA

### Extraction, sequencing, and analysis of genetic data

We extracted whole genomic DNA from tissue samples of four specimens (USNM 408876, UPMIN FDP_SGES_2208_088–UPMIN FDP_SGES_2208_090) using either the protocols in Weigt et al. (2012) or an AutoGenPrep 965 automated DNA extraction robot, following the manufacturer’s protocols. The barcode region of COI was sequenced from USNM 408876 following the protocols and primers of Baldwin et al. (2009). For USNM 408876, the barcode sequence contig was built, edited, and assembled using Geneious Prime v. 2024.0 and submitted to GenBank (PZ205242, Table 2). For UPMIN FDP_SGES_2208_088–UPMIN FDP_SGES_2208_090, whole mitochondrial genomes were captured using low-depth whole-genome sequencing, commonly called “genome skimming” (Hoban et al. 2022). Libraries were prepared using the NEB Ultra II FS DNA library prep kit and iTru y-yoke adapter and dual indices (Glenn et al. 2019). Pooled libraries were sent to Admera Health Biopharma Services (New Jersey, USA) for sequencing using an Illumina NovaSeq X Plus 10B in a paired-end 150-base-pair framework. Raw reads were uploaded to GenBank SRA (SRR37294250–SRR37294252, Table 2). Raw reads were cleaned of adapters and low-quality base pairs using fastp (Chen et al. 2018). Mitochondrial genomes were assembled using MitoFinder (Zhu et al. 2023) and annotated using MitoAnnotator (Iwasaki et al. 2013). Annotated mitogenomes were submitted to GenBank (PZ024373–PZ024375, Table 2). For UPMIN FDP_SGES_2112_020A and UPMIN FDP_SGES_2407_80A–UPMIN FDP_SGES_2407_80M, samples were processed following Fortaleza et al. (2025) with the primers FISH-COI-HBC/LBC (Weigt et al. 2012), and amplification success verified by agarose gel electrophoresis. PCR amplicons were sent to Macrogen (Korea) for enzymatic purification and bidirectional sequencing. A sequence contig was built, edited, and assembled using Geneious Prime v. 2023.0.4 following Fortaleza et al. (2025) and submitted to GenBank (OR524370 and PX978870–PX978858, Table 2).

To compare our sequence data to those on public databases, we searched GenBank and the Barcode of Life Database (BOLD) for platycephalids. We downloaded a subset of these sequences (Table 2) that were then combined with the newly generated sequences into a single alignment using MAFFT v. 7 (Katoh and Standley 2013). All mitochondrial genomes were trimmed to only include the orthologous base pairs for COI barcodes. The alignment was partitioned based on codon position and analyzed in a maximum-likelihood framework using IQ-TREE v. 3.0.1 (Chernomor et al. 2016; Kalyaanamoorthy et al. 2017; Wong et al. 2025), with the software determining the optimal models associated with each partition (i.e. MF; partition 1=TN+F+I+G4; partition 2=TN+G4; partition 3=HKY+F+I). Ten tree searches were performed, with support for the resulting topology assessed by generating 500 bootstraps (-bo). Analyses were rooted on *Scorpaenopsis cotticeps* Fowler, 1938.

## Results

### Family Platycephalidae

Taxon classificationAnimaliaPerciformesPlatycephalidae

4B88EBED-175F-5940-A565-84C32C63FB82

#### English name.

Flatheads.

#### Tagalog name.

Isdang-buwaya, isdambuwaya.

#### Bisaya name.

Sunogan, Sunug, Sunugun.

### Elates

Taxon classificationAnimaliaScorpaeniformesPlatycephalidae

Jordan & Seale, 1907

988CE14D-58B7-5930-9C2C-601FEC8D738A

#### Synonymy.

*Elates* Jordan & Seale, 1907 (type species *Elates thompsoni* Jordan & Seale, 1907, type by original designation and monotypy).

*Hyalorhynchus* Ogilby, 1910: 118 (type species *Hyalorhynchus pellucidus* Ogilby, 1910, type by monotypy).

#### Revised diagnosis.

A genus of platycephalid with a single preopercular spine, more than one vomerine tooth patch, no interopercular flap, and a caudal fin with the upper lobe more elongate than the lower lobe.

#### Remarks on *Elates ransonnettii*.

**Figure 2.**
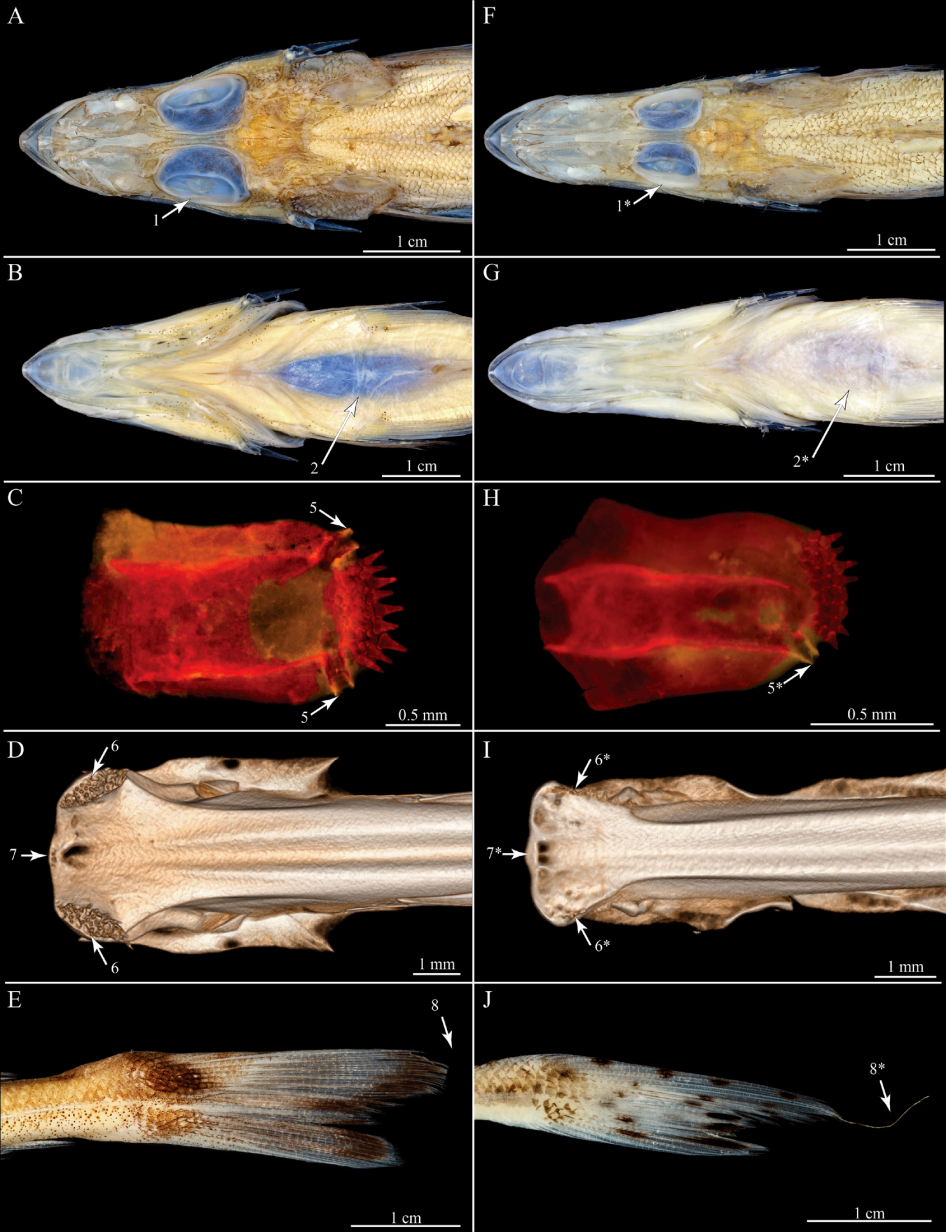
Morphological differences between *Elates saranganiensis* sp. nov. (**A–E)** and *E. ransonnettii* (**F–J**). Numbers without asterisks reflect characters of *E. saranganiensis* sp. nov. and with asterisks reflect characters of *E. ransonnettii*; **A**, **F**. Head in dorsal view. Characters: (1) larger eye and (1*) smaller eye; **B, G**. Body in ventral view. Characters: (2) dark ventral abdominal coloration and (2*) light ventral abdominal coloration; **C, H**. Alizarin-stained lateral-line scales in lateral view autofluorescing under Royal Blue LED light (see Materials and methods). Characters: (5) two lateral-line-scale pores and (5*) one lateral-line-scale pore; **D, I**. Vomer in ventral view imaged using µCT-scanned specimens. Characters: (6) two broad lateral vomerine tooth patches and (6*) two narrow vomerine tooth patches, (7) medial vomerine tooth patch and (7*) no medial vomerine tooth patch; **E, J**. Caudal fins with elongate upper lobes in lateral view. Characters: (8) no filamentous ray extends from upper caudal fin lobe and (8*) filamentous ray extends from upper caudal fin lobe. **A, B, D**. USNM 408876 124.2 mm SL; **C**. UPMIN FDP_SGES_2208_090, 151.4 mm SL; **E**. UPMIN FDP_SGES_2208_089, 197.4 mm SL; **F–H**. USNM 424737, 138.0 mm SL; **I**, **J**. USNM 437616, 137.0 mm SL.

**Figure 3.**
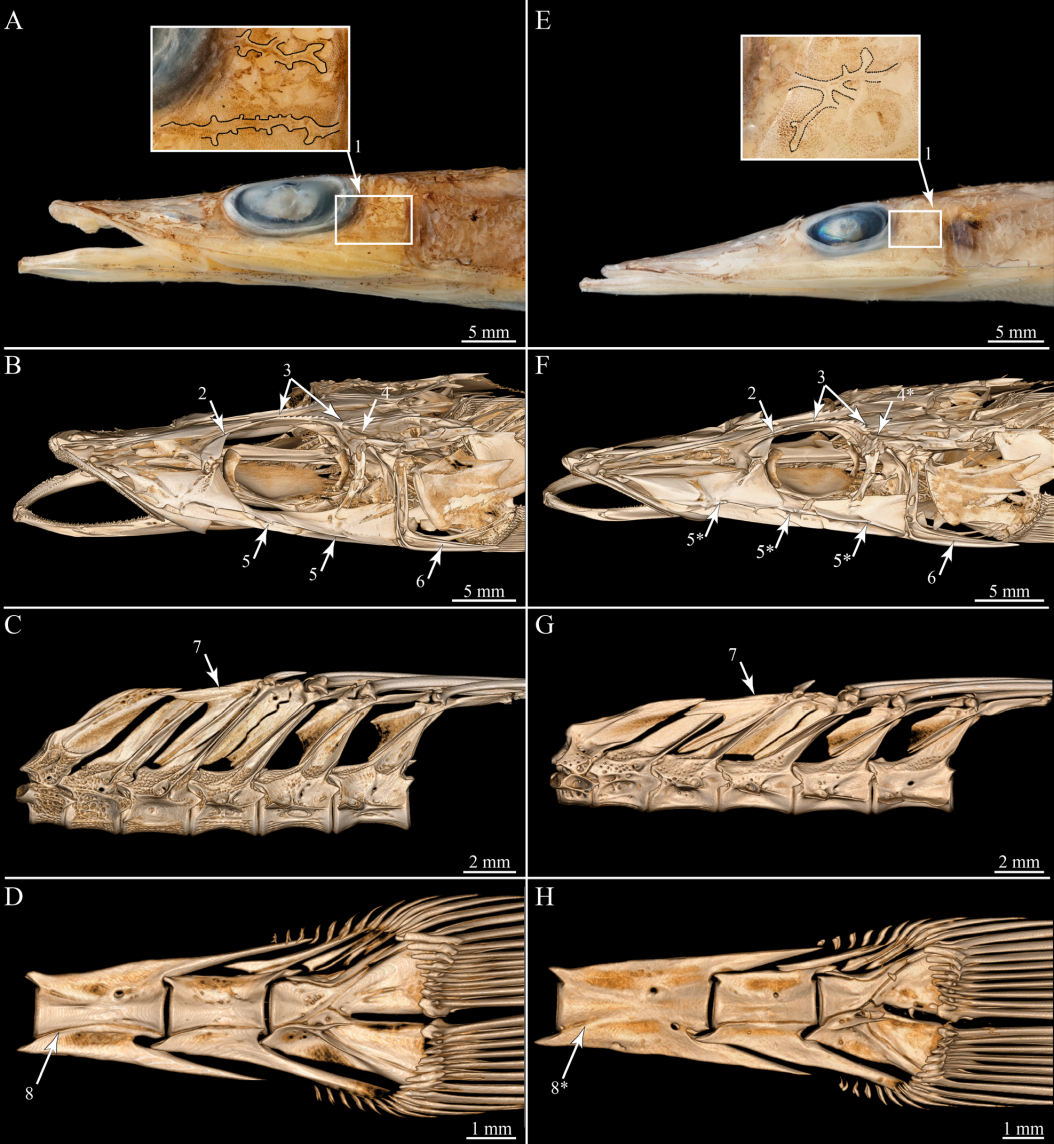
New data on anatomical characters referenced by Imamura (1996). *Elates saranganiensis* sp. nov. (**A–D**) and *E. ransonnettii* (**E–H**); **A, E**. Head in lateral view imaged using µCT-scanned specimens. Characters: (1) sensory tubes on cheek; **B**, **F**. Skull in a dorsolateral view imaged using µCT-scanned specimens with arrows indicating externally visible spines. Characters: (2) preocular spine, (3) supraorbital spines, (4) postocular spine absent and (4*) postocular spine present on extrascapular, (5) two suborbital spines and (5*) three suborbital spines, and (6) preopercular spine. Note that we observed variable presence, position, and sizes of these spines between specimens of the same species but spines highlighted represent the typical condition of these species; **C, G**. Spinous dorsal fin supports and anterior vertebrae in lateral view imaged using µCT-scanned specimens. Characters: (7) anterior most pterygiophore with dorsal-fin spine absent; **D, H**. Caudal skeleton in lateral view imaged using µCT-scanned specimens. Characters: (8) third preural centrum not fused with hemal spine and (8*) third preural centrum fused with hemal spine. **A**. UPMIN FDP_SGES_2208_090, 151.4 mm SL; **B–D**. USNM 408876, 124.2 mm SL; **E**. USNM 477242, 156.0 mm SL; **F–H**. USNM 437616, 137.0 mm SL.

Jordan and Seale (1907) described a filamentous ray extending from the upper lobe of the caudal fin in *E. ransonnettii* but did not note if this character demonstrated sexual or ontogenetic variation. We document that the filamentous ray extends from the upper lobe of the caudal fin in male, female, and juvenile specimens of *E. ransonnettii* (Fig. 2J). We also observed several characters previously undescribed from *E. ransonnettii*, including the presence of thin sensory tubes on the cheek (Fig. 3A) and, although there are usually six dorsal-fin spines, occasionally there is a seventh dorsal-fin spine present (Table 3). This differs from the observations of Imamura (1996) that *E. ransonnettii* has no sensory tubes on its cheek and that of Knapp (1999) and Imamura (2006) that *E. ransonnettii* has six dorsal-fin spines.

**Table 3.** Counts and measurements of type specimens for *Elates saranganiensis* sp. nov. with *E. ransonnettii* for comparison. Modes of meristic characters presented in parentheses where applicable.

Character	Holotype	Paratypes, *n* = 3	Non types, *n* = 14	*E. ransonnettii, n* = 23
Standard length (SL) (mm)	151.4	124.2–197.4	178.3–259.4	84.0–157.7
Total length	172	142–223.8	203.4–294.7	96.0–181
First dorsal-fin spines	I, VI	I, VI	I, VI	I, V–I, VI (I, V)
Second dorsal-fin rays	13	13	13	13
Anal-fin rays	13	13	13	13
Pectoral-fin rays	17	17–18 (17)	17–18 (18)	16–20 (17)
Pelvic-fin rays	I, 5	I, 5	I, 5	I, 5
Principal caudal-fin rays	15	15	15	14–16 (15)
Pored scales in lateral line	86	87–92	80–93	84–97 (93)
Longitudinal scale rows	96	90–106	90–106 (99)	99–117 (110)
Scales above lateral line	11	10–12	10–12 (11)	6–8 (6)
Gill rakers (upper + lower limbs)	6 + 14	6 + 14–6 + 15 (6 + 14)	6 + 14	6 + 14–6 + 15 (6 + 14)
Head length (% SL)	34.8	32.2–35.3	30.6–34.0	27.8–35.8
Pre-dorsal length (% SL)	36.6	35.5–37.4	33.0–36.3	30.6–36.5
First dorsal-fin base length (% SL)	16.6	11.4–16.6	13.4–16.1	10.0–15.3
Second dorsal-fin base length (% SL)	35.2	34.7–36.1	29.7–35.0	31.7–41.1
Anal-fin base length (% SL)	36.4	35.0–38.2	31.0–36.2	39.0–46.3
Caudal peduncle length (% SL)	6.8	8.0–8.7	8.9–10.5	7.4–11.9
Caudal peduncle depth (% SL)	2.8	2.6–2.7	2.3–3.0	2.7–3.9
Pectoral fin length (% SL)	15.2	14.2–16.4	15.2–17.8	12.3–19.4
Pelvic fin length (% SL)	16.1	14.9–17.3	14.3–17.5	13.4–19.5
Caudal fin length (% SL)	13.6	12.6–13.6	10.4–14.7	14.4–21.5
First dorsal-fin spine length (% SL)	0.9	0.8–1.0	0.7–1.1	0.5–2.0
Second dorsal-fin spine length (% SL)	14.6	12.5–14.1	11.6–14.1	10.9–15.5
First dorsal-fin ray length (% SL)	14.1	12.8–13.5	7.6–10.8	10.5–14.8
First anal-fin ray length (% SL)	9.3	8.2–11.3	6.6–9.0	7.2–12.2
Snout length (% HL)	37.7	32.75–37.0	38.8–42.7	37.6–43.8
Orbit diameter (% HL)	23.2	21.2–24.8	18.5–26.3	15.8–20.7
Preopercular spine length (% HL)	22.2	18.1–21.1	19.8–24.4	18.9–26.1
Upper jaw length (% HL)	32.1	30.3–35.3	33.1–35.8	27.9–37.3
Lower jaw length (% HL)	28.5	29.5–33.2	27.3–34.4	34.8–42.7
Interorbital width (% HL)	3.6	2.0–3.9	2.5–4.1	2.9–5.2
Postorbital length (% HL)	40.0	38.5–40.3	41.0–45.7	39.3–46.8
Suborbital width (% HL)	3.7	2.8–4.5	4.6–6.6	4.3–7.5

### Elates saranganiensis sp. nov.

Taxon classificationAnimaliaScorpaeniformesPlatycephalidae

BD9FDF8D-E419-5B5C-86E5-B5DA3D55FE10

https://zoobank.org/28BCF1FD-C82E-4883-9E0D-EBD103002CC6

#### English name.

Sarangani Flathead.

#### Tagalog name.

Isdang-buwaya ng Sarangani, isdambuwaya ng Sarangani.

#### Bisaya name.

Sunogan sa Sarangani.

#### Type materials.

All type and non-type specimens were purchased from General Santos Public Market, General Santos City, South Cotabato, Philippines (6.108°N, 125.179°E). The capture locality, as reported by vendors, is included when available.

**Figure 4.**
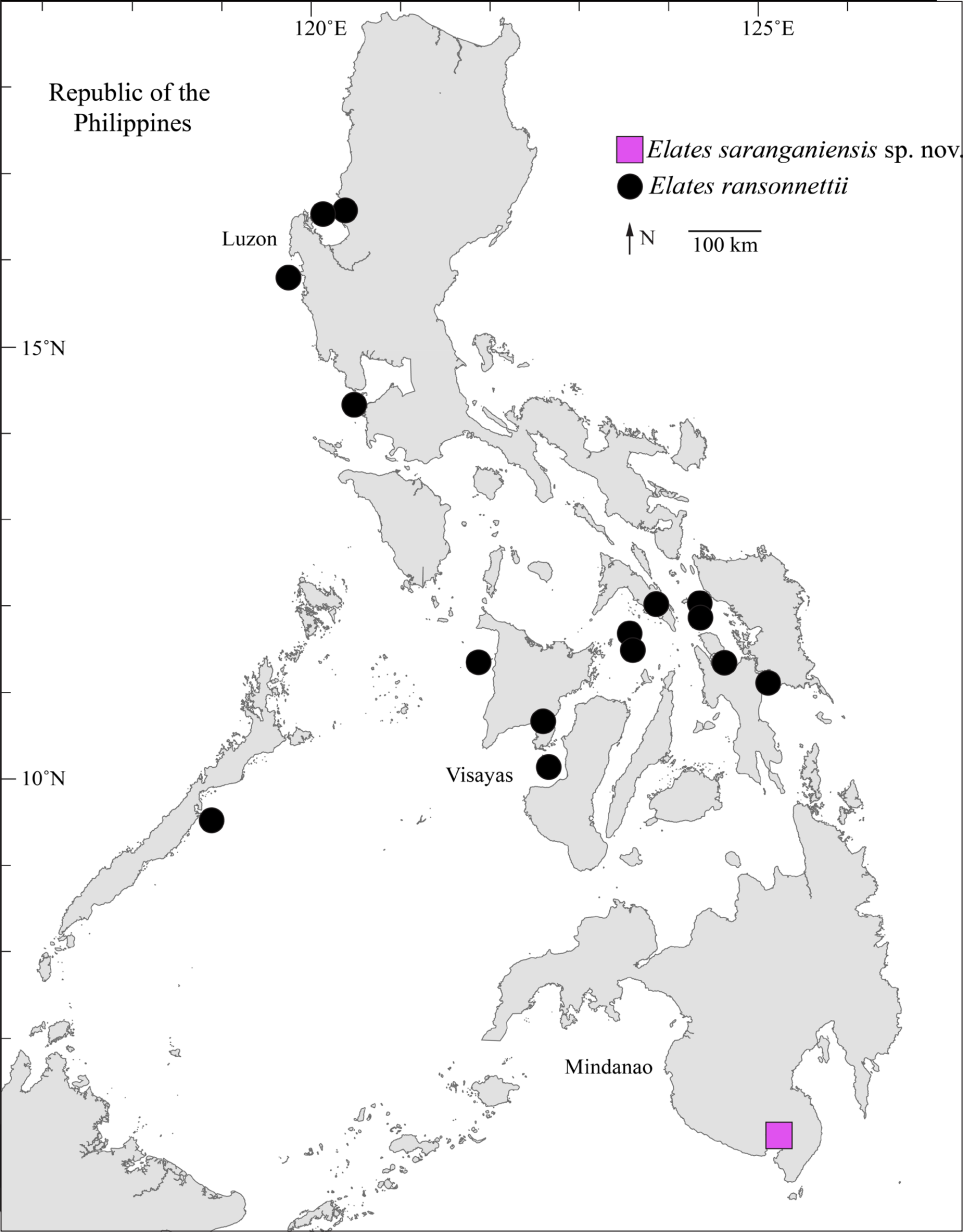
Distribution of the genus *Elates* in the Philippines. All specimens of *E. saranganiensis* sp. nov. are represented by the purple square over the General Santos Public Market. Localities for USNM specimens of *E. ransonnettii* are represented by black circles.

**Figure 5.**
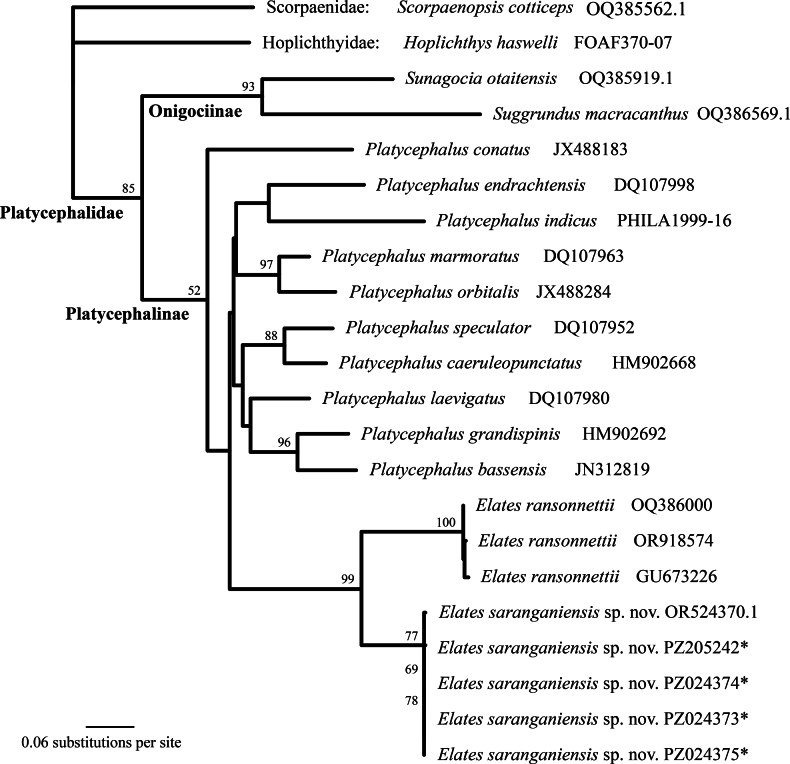
Phylogenetic relationships of *Elates* and other members of Platycephalidae based on the barcode region of cytochrome c oxidase subunit I. GenBank numbers listed after taxon except for *Hoplichthys haswelli* and *Platycephalus indicus*, as they are only represented by a BOLD identifier. “*” indicates type material. Bootstrap values based on 500 standard replicates, values < 50% not shown.

***Holotype***. University of the Philippines Mindanao (UPMIN) FDP_SGES_2208_090 (Figs 1, 2, 3, 4, 5, Tables 2, 3), 154.1 mm SL; purchased 2 Aug. 2022; MA Fortaleza, JJ Lanutan leg. ***Paratypes***. USNM 408876 (Figs 1, 2, 3, 4, 5, Tables 2, 3), 124.2 mm SL; purchased 21 May 2012; vendors reported collection from the vicinity of Glan; Sarangani, MA Fortaleza, JJ Lanutan leg. • UPMIN FDP_SGES_2208_088 (Figs 3, 4, 5, Tables 1, 2); 180.0 mm SL; purchased 2 Aug. 2022; MA Fortaleza, JJ Lanutan leg. • UPMIN FDP_SGES_2208_089 (Figs 3, 4, 5, Tables 2, 3); 197.4 mm SL; purchased 2 Aug. 2022; MA Fortaleza, JJ Lanutan leg.

#### Non-type material.

Vendors reported collection from the vicinity of Buayan, General Santos City. UPMIN FDP_SGES_2407_80A to UPMIN FDP_SGES_2407_80M (Tables 2, 3); 13 specimens, 180.4–259.4 mm SL; purchased 22 July 2024; MA Fortaleza, JP Cabasan, JJ Lanutan, JAP Oño leg. • UPMIN FDP_SGES_2112_020A; purchased 12 Aug. 2021, 178.3 mm SL; CL Nañola, MA Fortaleza, JJ Lanutan, KL Labrador leg.

#### Synonymy.

*Elates ransonnettii* Fortaleza et al., 2025: 12 (noted BOLD:ACK4730 did not cluster with existing genetic data for the species).

#### Diagnosis.

A species of *Elates* with an eye-in-snout length of < 1.8 (Fig. 2A; > 2.1 in *E. ransonnettii*, Fig. 2F), lateral vomerine tooth patch broad (Fig. 2D; narrow in *E. ransonnettii*, Fig. 2I), medial vomerine tooth patch present (Fig. 2D; only visible in some specimens; absent in *E. ransonnettii*), lateral-line scales with two pores (Fig. 2C; one pore in *E. ransonnettii*, Fig. 2H), scale rows above the lateral line 10–12 (Fig. 2A; 6–8 in *E. ransonnettii*, Fig. 2F), filamentous ray on caudal fin absent (Fig. 2E; present extending from third caudal-fin ray in *E. ransonnettii*, although easily lost, Fig. 2J), spinous- and soft-dorsal-fin membrane transparent (Fig. 1B; spotted along spines and rays in *E. ransonnettii*, Fig. 1D), and peritoneum wholly dark (Fig. 2B; dorsally dark, ventrally pale in *E. ransonnettii*, Fig. 2G).

#### Description.

Counts and measurements listed in Table 3. Head large, body depressed anteriorly, with elongate caudal peduncle (Fig. 1A, B). First dorsal-fin spine small, not connected to other spines by membrane. Dorsal spines II–VII connected by membrane. Pored lateral-line scales 86 (87–92 in paratypes). Entire lateral line visible when viewed laterally; only anterior half of lateral line visible dorsally (Fig. 1A, B). Stout lateral-line-scale spines absent. Body scales mostly ctenoid with some cycloid ventrally. Opercle and preopercle scaled. Sensory tubes on cheek weakly branched, two patches posterior to the lower half of eye (Fig. 3A; moderate branching in FDP_SGES_2208_089). Head spines and ridges weakly developed, preopercular spine large (condition of spines in paratypes and non-type material varied in presence, size, and position). Postocular spine absent (Fig. 3B). Eyes large, iris lappet absent, and ocular flaps absent. Many rows of small teeth on maxilla, some on the palatine. Teeth along interior edges of maxillary and lateral vomerine tooth patches elongate, posteriorly directed. Crescent-shaped lateral vomerine tooth patch bearing many rows of teeth. Small medial vomerine tooth patch bearing few teeth. Dentary containing two main rows of teeth, with stouter teeth labially, a diastema separating labial and lingual rows, and rows of smaller teeth lingually. Coronomeckelian bone absent. Anterior-most pterygiophore not bearing a dorsal-fin spine (Fig. 3C). Dorsal-fin origin posterior to head. Pectoral-fin rays extending posteriorly to middle of spinous dorsal fin. Pelvic-fin rays extending beyond posterior margin of spinous dorsal fin, not reaching anal fin. Soft dorsal and anal fins falcate, with similar origins and terminations. Third preural centrum not fused with hemal spine (Fig. 3D). Caudal fin with upper lobe more elongate than lower lobe, lateral line extending onto the median caudal rays.

#### Life-like coloration of specimens.

Body gray-brown, dorsum spotted with dark, brown-black pigment and saddles present on the posterior end of dorsum (Fig. 1A; saddles absent in USNM 408876 and UPMIN FDP_SGES_2208_089). Dorsal- and anal-fin membranes transparent, with spines and rays ranging from transparent to brown. Pectoral fins gray-brown. Caudal fin with dark margin along distal end (oblong patches of dark pigment on lower lobe in USNM 408876). Ventral coloration not examined in fresh specimens.

#### Preserved coloration of specimens.

Body brown, dorsum spotted with brown-black pigment, faint saddles on the posterior end of dorsum (Fig. 1A; saddles absent in USNM 408876). Several elongate, dark blotches present along the ventral median edge of the preopercle. Small dark spots peppered along the underside of the lower jaw, under the posterior margin of the opercle, and encircling the base of the pectoral and pelvic fins. Black peritoneum visible externally on the ventral side, forming a dark oval with surrounding ventral coloration of brown and white. Dorsal-, anal-, pectoral-, pelvic-, and caudal-fin coloration as in life.

#### Etymology.

The specific name *saranganiensis* refers to the Sarangani Bay, from which the type specimens were captured.

#### Distribution, habitat, size, and fisheries.

All records of *E. saranganiensis* were purchased from the General Santos Public Market in the South Cotabato Province of Mindanao Island in the southern Philippines and were collected nearby in the Sarangani Bay (Fig. 4). The vendors of USNM 408876 stated that the fish was likely captured near Glan, Sarangani. Vendors of specimens UPMIN FDP_SGES_2407_80A to UPMIN FDP_SGES_2407_80M stated that they were collected in the vicinity of Buayan, at depths of 2–50 m over rocky and reef habitats. The vendors reported *E. saranganiensis* is less abundant during the rainy season, which occurs from June to September (de Jesus et al. 2001). The 18 specimens ranged from 124.2–259.4 mm SL (Table 3). The sex and maturity of the single *E. saranganiensis* we dissected (USNM 408876) was unable to be determined.

*Elates saranganiensis* is sold under the name sunogan, which means “browning” or “having brown discoloration”. Sunogan is not specific to *E. saranganiensis* and is used for other platycephalids, as well as other families of fishes like paralichthyids and fistulariids (Herre and Umali 1948; Ganaden and Lavapie-Gonzales 1999). Vendors stated that *E. saranganiensis* is captured using longlining and bottom-set gillnets and is usually prepared fried, cooked in clear soup (tinola or sinabawan), or cooked in vinegar (paksiw).

#### Mitochondrial genome.

The mitochondrial genome of *Elates saranganiensis* is 16,610–16,611 base pairs in length and encodes 2 rRNAs, 13 protein-coding genes (PCGs), 22 tRNAs, and one non-coding control region. Locus order is as follows: tRNA-Phe | 12S rRNA | tRNA-Val | 16S rRNA | tRNA-Leu | ND1 | tRNA-Ile | tRNA-Gln | tRNA-Met | ND2 | tRNA-Trp | tRNA-Ala | tRNA-Asn | tRNA-Cys | tRNA-Tyr | COI | tRNA-Ser | tRNA-Asp | COII | tRNA-Lys | ATPase8 | ATPase6 | COIII | tRNA-Gly | ND3 | tRNA-Arg | ND4L | ND4 | tRNA-His | tRNA-Ser | tRNA-Leu | ND5 | ND6 | tRNA-Glu | Cytb | tRNA-Thr | tRNA-Pro | D-loop. Twenty-nine loci, including 2 rRNAs, 12 PCGs, 14 tRNAs, and the control region occur on the H-strand, with ND6 and 8 tRNAs occurring on the L-strand.

#### Phylogenetic analysis.

Our analyses of the barcode region of COI resulted in a single optimal topology with a score of –4270.842. All samples of *E. saranganiensis* were recovered in a clade sister to a clade of all sequences of *E. ransonnettii* sampled (Fig. 5). The clade containing the two species of *Elates* was recovered as sister to a clade including all sampled species of *Platycephalus* except for *P. conatus*, which is recovered as sister to the clade of all species of *Elates* and *Platycephalus* analyzed (Fig. 5).

## Discussion

Imamura (1996) diagnosed the genus *Elates* as having a narrow head and body, two suborbital spines present below the middle and posterior section of each eye, two vomerine tooth patches, preopercle with a single long spine, lateral-line scales with one opening posteriorly, no interopercular flap (see Imamura 1996: fig. 51), and undeveloped sensory tubes on the cheek. With the description of a second species, *E. saranganiensis*, which has two pores on the posterior end of the lateral-line scales, two suborbital spines that are not always aligned with the median and posterior of the eye, and a broader body, we revise the diagnosis for the genus *Elates* (see Revised diagnosis above). Beyond these characters, we remove the absence of sensory tubes on the cheek from the generic diagnosis because we identified these structures in both *E. saranganiensis* and *E. ransonnettii* (Fig. 3). Imamura (1996: fig. 53) illustrated sensory tubes on the cheeks of other platycephalids ventral to the suborbital ridge in fig. 53, while those we identified are on the dorsal portion of the cheek (Fig. 3). Finally, we include the elongate upper caudal-fin lobe in our diagnosis. First noted as a generic character by Jordan and Seale (1907) but not included in the diagnosis by Imamura (1996), we found this character useful to distinguish *Elates* from other platycephalid genera. With the results of our phylogenetic analysis (Fig. 5) recovering *E. saranganiensis* and *E. ransonnettii* as sister taxa, we take a conservative approach and place the new species in the genus *Elates* rather than describe a new monotypic genus. The two species of *Elates* share several morphological features that are absent in species of *Platycephalus*, including a single preopercular spine, multiple patches of vomerine teeth, and absence of a coronomeckelian bone. However, there are several shared characters among *E. saranganiensis* and species of *Platycephalus*, such as the broader shape of the body, reduction in number of lateral-line scales, and lack of fusion between the third preural centrum and hemal spine (Fig. 3D). Additionally, the median vomerine tooth patch of *E. saranganiensis* could be homologous to the united vomerine tooth patch present in species of *Platycephalus*. Investigations into the taxonomy and phylogeny of the subfamily are needed to understand the monophyly of *Platycephalus*, with *P. conatus* recovered in a separate clade from the rest of the sampled *Platycephalus* in our phylogeny (Fig. 5). The discovery and description of more taxa and further investigation into the subfamily Platycephalinae may warrant the description of a separate genus for *E. saranganiensis* in the future.

The largest specimen of *E. saranganiensis* examined was 294.7 mm total length (TL), which exceeds the maximum reported size of *E. ransonnettii* at 190 mm TL (Knapp 1999), suggesting that *E. saranganiensis* is a larger species. Information about the maturity and reproduction of *E. saranganiensis* remains unknown. Given that the smallest mature *E. ransonnettii* we dissected were approximately 100 mm SL and the larger size of *E. saranganiensis*, the new species likely reaches maturity at lengths greater than 100 mm SL.

We examined more than 1300 lots of platycephalids in the USNM collection but did not find evidence that *E. saranganiensis* has been collected outside of Sarangani Bay. Thus, it may be endemic to this region. If the range of *E. saranganiensis* extends elsewhere, it is likely further south along the relatively shallow waters of the Sangihe Islands or the Davao Gulf. Specimens were captured near the mouths of the Glan and Buayan Rivers. While much of the Sarangani Bay is nearly oceanic in salinity (de Jesus et al. 2001), these river mouths provide estuarine waters, which are a common habitat for other species of Platycephalinae (Knapp 1999). Changes in abundance reported by vendors may suggest that individuals move to another area seasonally. Given its limited distribution, this new species may warrant conservation concern, and we recommend additional study.

## Supplementary Material

XML Treatment for Platycephalidae

XML Treatment for Elates

XML Treatment for Elates saranganiensis

## Additional information

### Artificial Intelligence (AI) use

The authors accept full responsibility for the content of the manuscript, including the disclosure of any use of AI.

No AI tools were used in the preparation of this manuscript.

### Author ORCIDs

N.S. Rose https://orcid.org/0009-0003-2273-9551

M.A. Fortaleza https://orcid.org/0000-0003-0022-5333

J.P. Cabasan https://orcid.org/0000-0001-5472-6729

K.L. Labrador https://orcid.org/0000-0002-3309-8062

J.J. Lanutan https://orcid.org/0000-0002-1387-5346

C.L. Nañola Jr. https://orcid.org/0000-0002-1563-0369

M.G. Girard https://orcid.org/0000-0003-3580-6808

K.E. Bemis https://orcid.org/0000-0002-7471-9283

### Data availability

All data supporting this study are available in the paper, in GenBank, in BOLD, or in MorphoSource.
